# Role of NHERF1 in MicroRNA Landscape Changes in Aging Mouse Kidneys

**DOI:** 10.3390/biom14091048

**Published:** 2024-08-23

**Authors:** Anish Jain, Hyun Jun Jung, Joseph Aubee, Jahn N. O’Neil, Laila A. Muhammad, Shaza Khan, Karl Thompson, Maurice B. Fluitt, Dexter L. Lee, Carolyn M. Klinge, Syed J. Khundmiri

**Affiliations:** 1 Department of Physiology, Howard University College of Medicine, Washington, DC 20059, USA; ajain8@stanford.edu (A.J.); jahn.oneil@howard.edu (J.N.O.); laila.muhammad@bison.howard.edu (L.A.M.); shaza.khan@nih.gov (S.K.); dllee@howard.edu (D.L.L.); 2 Division of Nephrology, Department of Medicine, Johns Hopkins University School of Medicine, Baltimore, MD 21205, USA; hjung24@jhmi.edu; 3 Department of Microbiology, Howard University College of Medicine, Washington, DC 20059, USA; joseph.aubee@gmail.com (J.A.); karl.thompson@howard.edu (K.T.); 4 Department of Medicine, Howard University College of Medicine, Washington, DC 20059, USA; maurice.fluitt@howard.edu; 5 Department of Biochemistry and Molecular Genetics, University of Louisville School of Medicine, Louisville, KY 40202, USA; carolyn.klinge@louisville.edu

**Keywords:** sodium-hydrogen exchanger regulatory factor-1, kidneys, aging, miRNA, miR-153, nuclear factor of activated T-cells (NFATc2/3)

## Abstract

MicroRNAs (miRNAs) play important roles in the regulation of cellular function and fate via post-transcriptional regulation of gene expression. Although several miRNAs are associated with physiological processes and kidney diseases, not much is known about changes in miRNAs in aging kidneys. We previously demonstrated that sodium hydrogen exchanger 1 (NHERF1) expression regulates cellular responses to cisplatin, age-dependent salt-sensitive hypertension, and sodium-phosphate cotransporter trafficking. However, the mechanisms driving these regulatory effects of NHERF1 on cellular processes are unknown. Here, we hypothesize that dysregulation of miRNA-mediated gene regulatory networks that induce fibrosis and cytokines may depend on NHERF1 expression. To address this hypothesis, we compared miRNA expression in kidneys from both male and female old (12–18-month-old) and young (4–7-month-old) wild-type (WT) and NHERF1 knockout (NHERF1^−/−^) mice. Our results identified that miRNAs significantly decreased in NHERF1^−/−^ mice included miR-669m, miR-590-3p, miR-153, miR-673-3p, and miR-127. Only miR-702 significantly decreased in aged WT mice, while miR-678 decreased in both WT and NHERF1^−/−^ old versus young mice. miR-153 was shown to downregulate transcription factors NFATc2 and NFATc3 which regulate the transcription of several cytokines. Immunohistochemistry and western blotting revealed a significant increase in nuclear NFATc2 and NFATc3 in old NHERF1^−/−^ mice compared to old WT mice. Our data further show that expression of the cytokines IL-1β, IL-6, IL-17A, MCP1, and TNF-α significantly increased in the old NHERF1^−/−^ mice compared to the WT mice. We conclude that loss of NHERF1 expression induces cytokine expression in the kidney through interactive regulation between miR-153 and NFATc2/NFATc3 expression.

## 1. Introduction

Aging is the progressive deterioration of the structure and function of cells, tissues, and organs over time. Cells experience increasing levels of senescence and lose the ability to grow and divide, resulting in tissue and organ dysfunction. Physiological aspects of aging include decreased cardiac output, hypertension, and atherosclerosis [1]. Aging is a normal process of life and is highly heterogeneous between individuals. Studies in animal models of aging such as Fisher-344 (F344) and Fisher-Brown Norway (FBN) rats suggest differences between the two species. F344 rats develop chronic kidney disease and have increased levels of cytokines in the kidneys while the FBN rats age healthily without signs of chronic diseases [2,3,4]. Using proteomics, our laboratory has recently demonstrated that the F344 rats lose sodium hydrogen regulatory factor 1 (NHERF1) expression in the kidneys during aging [5]. While the F344 rats have been shown to develop chronic kidney disease with age, they are salt insensitive and do not develop hypertension from increased dietary salt intake [6]. In contrast, the FBN rats showed increased NHERF1 expression with age and developed hypertension in response to increased dietary salt intake [7].

NHERF1 is a PDZ-binding protein with two class I PDZ domains that bind to several ion transporters and G-protein-coupled receptors. Our laboratory demonstrated a role for NHERF1 in the trafficking of sodium phosphate cotransporter in the kidney proximal tubules and renal response to cisplatin [8,9]. We have also demonstrated that a lack of NHERF1 expression prevents salt-sensitive hypertension [7]. However, the mechanisms or pathways in these processes are not well understood.

Epigenetic mechanisms of gene regulation are increasingly being recognized as having a major influence on the pathogenesis of kidney diseases. Non-coding RNAs like microRNAs (miRNA) represent an intricate mode of epigenetic regulation. MicroRNAs (miRNAs) are small 22 nucleotide single-stranded RNA molecules that regulate transcription and translation of proteins [10,11]. MicroRNAs act to regulate gene expression at the post-transcriptional level, often binding to the 3′ untranslated region of their target mRNAs through complementary base pairing [11]. The dysregulation of miRNAs is associated with several pathophysiological conditions such as renal fibrosis, hypertension, and chronic kidney failure. Changes in miRNA-21, -29, -192, and -200 levels are associated with TGF-β-mediated renal fibrosis [12,13,14]. In patients with essential hypertension, miR-505 levels are consistently higher [15]. The expression of miR-21, miR-122, miR-637, and miR-let-7e have been reported to be higher in hypertensive patients [15]. Expression of several miRNAs including miRNA-320, miR-26b, and miR-21 have been documented in the arteries of the hypertensive Dahl salt-sensitive (DSS) rat model. However, very few studies are reported in the literature showing changes in aging kidneys. Kwekel et al. [2] performed an extensive study to determine changes in expression of miRNA in aging kidneys using the F344 rat model. They showed that differential expression of miR-214, miR-130b, miR-150, miR-223, miR-142-5p, miR-185, and miR-296 during aging correlates with renal inflammation and nephritis. Similarly, several miRNAs including miR-21, miR-146a, and miR-199a have been identified as markers of renal ischemia reperfusion [16,17,18,19].

Although pathological changes in the kidney during aging are more prominent in the renal cortex, most miRNA studies have used the whole kidney. The goal of this observational study was to identify global changes in miRNA levels during aging and the role of NHERF1 in regulation of miRNA expression in the kidneys. To address this aim, we analyzed differential expression of miRNAs in whole kidneys from young and old NHERF1^−/−^ mice and their WT littermates. Our data demonstrated a decrease in expression of miRNA-153 in old NHERF1^−/−^ mice, leading to nuclear expression of NFATc2 and NFATc3, transcription factors that regulate the expression of cytokines including IL-6. These data suggest a role for NHERF1 expression in the regulation of miRNA expression in aging kidneys.

## 2. Materials and Methods

Animals: All the animal experiments were performed according to the US Guide for the Care and Use of Laboratory Animals and approved by the Institutional Animal Care and Use Committee (IACUC) at Howard University (IACUC-MED-15-07) and the University of Louisville (IACUC # 13129). NHERF1^−/−^ mice 1–4 months (young, *n* = 6, 3 males and 3 females) and 12–24 months (*n* = 6, 3 males and 3 females) and their age-matched wild type (WT) littermates (*n* = 12, 6 males and 6 females) on C57Bl/6J background were provided by Dr. Lederer (University of Texas Southwestern, Dallas, TX, USA). The animals were stabilized on standard rodent chow and water ad libitum for one week prior to the experiments. The animals were euthanized under 2% isoflurane, and the kidneys were collected, decapsulated, and immediately frozen in liquid nitrogen and stored at −80 °C ultra-freezer until RNA isolation.

miRNA Isolation: Total small RNAs were isolated from whole kidneys using Ambion miRVana kit (Cat. # AM1560—Thermo Fisher, Waltham, MA, USA) according to the manufacturer’s protocol. The mirVana selects for RNA species smaller than 200 nucleotides. Briefly, whole kidneys were homogenized in lysis/binding buffer (10 mL/mg tissue). A 1/10th volume of miRNA additive was added to the homogenates to enrich small RNAs followed by extraction in acid-phenol chloroform. Total RNA was eluted in RNase-free water heated to 95 °C. RNA concentration was measured using NanoDrop8000 (Thermo Fisher, Waltham, MA, USA).

miRNA Identification: 20 ng/μL RNA was used to identify differentially expressed miRNAs on NanoString platform using nCounter mouse v1.5 miRNA assay (CSO-MMIR15-12) from NanoString (Seattle, WA, USA). The high throughput nCounter multiplexed panel identifies over 577 miRNAs from tissue samples without the need for reverse transcription and amplification. Specimen preparation, annealing, ligation, and purification were performed according to the manufacturer’s guidelines and as described by Aravindraja et al. [20].

Analysis of miRNA Profiles: miRNA profiles were analyzed first by using nSolver analysis software 4.0 from NanoString (Seattle, WA, USA). Raw data generated by nSolver were normalized based on the top 100 miRNA genes expressed. Briefly, the QC was carried out following the manufacturer’s guidelines and as described by Aravindraja et al. [20]. Only samples that passed the QC were included in the data analysis. To reduce the background/noise ratio, a background threshold value, calculated as average of eight negative control values from all samples, was set to 22.67. The normalized data (log10 of the original values) are shown in Supplementary Table S1. The raw data files, generated after passing the QC, have been uploaded to the Mendeley Data sharing platform (https://data.mendeley.com/drafts/xkp9dy9z4f, Reserved https://doi.org/10.17632/xkp9dy9z4f.1). We did not observe sex differences in any of the groups and therefore data from male and female animals were pooled for analysis. The changes in miRNA between the groups were analyzed for statistical changes using GraphPad software (GraphPad Prism Version 10.2). A *p*-value less than 0.05 by student’s *t*-test followed by Bonferroni correction was used as an a priori for significant change.

In silico miRNA network analysis: We performed pathway and network analysis of differentially expressed miRNAs in MetaCore^TM^ version 20.3 (GeneGO, Thomson Reuters, New York, NY, USA) as described by Schultz et al. [21]. The significantly different miRNAs were included in the analysis by MetaCore^TM^. MetaCore^TM^ is a web-based software suite for multiple applications in systems biology including the miRNA-seq analysis as used here. For each analysis, the appropriate miRNA data species, i.e., Mus musculus and Rattus norvegicus, was used. MetaCore^TM^ analyses are based on MetaBase (http://metadatabase.org/, accessed on 24 September 2020), a 100% manually curated integrated database that contains over 2.2 million experimental findings on mammalian biology from PubMed records (https://pubmed.ncbi.nlm.nih.gov/, accessed on 24 September 2020) on protein–protein, protein–DNA, protein–RNA, and protein–compound interactions; metabolic and signaling pathways; and other information. All MetaCore analyses are uploaded to the Mendeley data-sharing site (https://data.mendeley.com/drafts/xkp9dy9z4f, Reserved https://doi.org/10.17632/xkp9dy9z4f.1). 

Western blotting: Kidney homogenates were centrifuged at 2500× *g*, and the crude nuclear pellet was resuspended in RIPA buffer. The homogenates were subjected to western blotting for NFAT2c using anti-NFATc2 antibodies (Cat. # 22023-1-AP, Proteintech, Rosemont, IL, USA) at 1:1000 dilution as described previously [22].

Immunohistochemistry: Paraffin-embedded kidney sections were subjected to IHC for NFATc2 (Cat. # 22023-1-AP) and NFATc3 (Cat. # 18222-1-AP) using antibodies from Proteintech (Rosemont, IL, USA) at 1:500 dilution as described previously [23].

Measurement of Cytokines: Cytokines, IL-1a (Cat. # BMS611), IL-6 (Cat. #A43656), IL-10 (Cat. #BMS614), IL-17A (Cat. #BMS6001), MCP-1 (Cat. #BMS6005), and TNF-α (Cat. #BMS607-3) levels were measured in kidney homogenates using mouse ELISA kits from Thermo Fischer (Waltham, MA, USA) according to the manufacturer’s protocol. In each assay, 50–100 μg homogenate protein (in 100 mM Tris-HCl, pH 7.4) was used to measure the cytokines.

**Figure 2:**
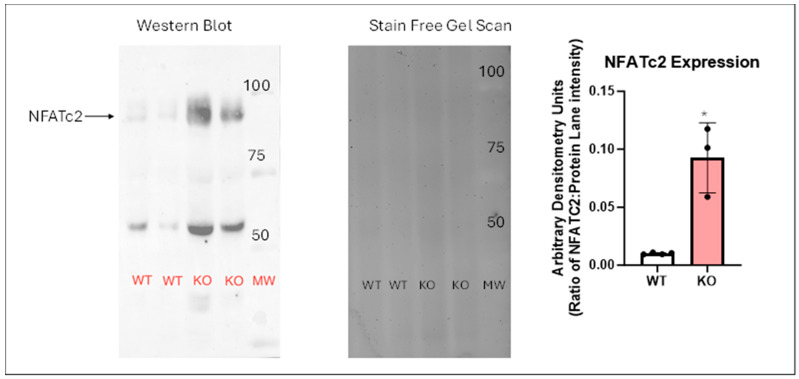
Expression of NFATc2 in the nuclear fragment of kidneys from WT and NHERF1^−/−^ mice. Nuclear fractions were prepared from kidney homogenates of 18–24-month-old WT or NHERF1^−/−^ mice as described in Materials and Methods. Proteins were separated by 10% SDS-PAGE followed by western blotting using an antibody against NFATc2 (**left panel**). A representative blot from kidneys of one male (lanes 1 and 3) and one female (lanes 2 and 4) and the corresponding gel image (stain free image, (**middle panel**)) are shown. The bar diagram (**right panel**) shows data as ratio of arbitrary band density to the density of the lane in stain free gel scan (Mean ± SD, *n* = 3). Data from each individual animal is denoted with a circle on the bar graph. * indicates *p* < 0.05 from WT kidneys. There were no significant sex differences between the groups, and therefore the data from both males and females were pooled together in the bar diagram. File S1: Spplementary Tables. Original images can be found in Figure S1.

**Figure 4:**
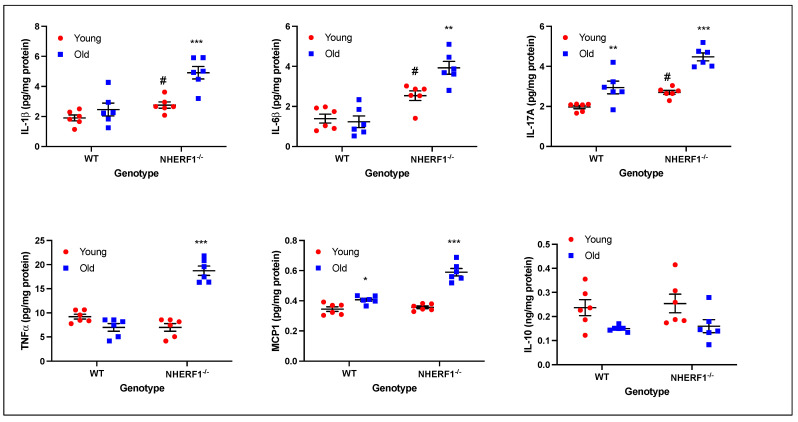
Expression of cytokines in kidneys from young and old WT and NHERF1^−/−^ mice. Kidney homogenates from 2–4-month or 18–24-month-old WT or NHERF1^−/−^ mice were subjected to ELISA for determination of IL-1, IL-6, IL-17, MCP-1, TNFα, or IL-10 levels. The data are presented as Mean ± SD from 6 (3 males and 3 females) different animals represented by each data point. # indicates *p* < 0.05 from respective WT age-group. *** *p* < 0.001 from NHERF^−/−^ young mice, * or ** indicate *p* < 0.05 or <0.01 from WT young mice respectively.

Statistical analysis: Statistical evaluation of the data was performed using GraphPad Prism (version 10.2). For individual miRNA comparisons, a two-tailed *t*-test was performed with *p* < 0.05 considered significant. Data in Figure 2 and Figure 4 was analyzed by two-way ANOVA followed by Bonferroni analysis.


**Table of Resources.**



**Reagent/Resource**

**Source**

**Cat. No.**
Ambion miRVana KitThermo Fisher, Waltham, MA, USAAM1560nCounter mouse v1.5 miRNA assayNanoString, Seattle, WA, USACSO-MMIR15-12MetaCore GeneGo, Thomson Reuters, New York, NY, USAVersion 20.3Anti-NFATc2Proteintech, Rosemont, IL, USA22023-1-APAnti-NFATc3Proteintech, Rosemont, IL, USA18222-1-APAnti-Rabbit-HRPCell Signalling Technology, Danvers, MA, USA7074SAnti-Rabbit BiotinylatedVector Laboratories, Newark, CA, USABA-1000-1.5VECTASTAIN^®^ Elite^®^ ABC Universal PLUS Kit, Peroxidase (Horse Anti-Mouse/Rabbit IgG)Vector Laboratories, Newark, CA, USAPK-8200DAB Substrate Kit, Peroxidase (HRP), with Nickel, (3,3′-diaminobenzidine)Vector Laboratories, Newark, CA, USASK-4100IL-1aThermo Fisher, Waltham, MA, USABMS611IL-6Thermo Fisher, Waltham, MA, USAA43656IL-10Thermo Fisher, Waltham, MA, USABMS614IL-17AThermo Fisher, Waltham, MA, USABMS6001MCP-1Thermo Fisher, Waltham, MA, USABMS6005TNF-alphaThermo Fisher, Waltham, MA, USABMS607-3All other ChemicalsMillipore-Sigma, St. Louis, MO, USA


## 3. Results

Effect of age on miRNA profile in NHERF1^−/−^ mice: To determine the changes in the miRNA profile in the kidneys between young and old WT or NHERF1^−/−^ mice, we isolated the total mRNA and determined the changes in the miRNA profile on the NanoString platform. We considered the significantly changed miRNAs with at least 30% change in either direction with a *p*-value less than 0.05. First, we analyzed the miRNA differences between young WT and NHERF1^−/−^. As shown in Table 1, three miRNAs, miR-691, miR-291b, and miR18b, were decreased significantly in the young (2–4-month-old) NHERF1^−/−^ mice as compared to the age-matched WT mice.

Next, we analyzed the differences between 12–18-month-old WT and NHERF1^−/−^ mice. As shown in Table 2, 13 miRNAs significantly decreased in NHERF1^−/−^ mice as compared to the age-matched WT mice including miR-92 which has a poorly conserved site on position 79–85 in the 3′ UTR of NHERF1 gene (https://www.targetscan.org/cgi-bin/targetscan/mmu_80/view_gene.cgi?rs=ENSMUST00000021077.3&taxid=10090&members=miR-92a-2-5p&showcnc=1&shownc=1&shownc_nc=1&showncf1=1&showncf2=1&subset=1, (accessed on 31 January 2024)).

To determine age-dependent changes in kidney miRNA expression, we analyzed the data between the young and old WT mice. As shown in Table 3 and Table 5, the miRNAs were increased and the 19 miRNAs were decreased in the old WT mice as compared to the young WT mice. The top hit mir-1186 is a dead hairpin loop entry in the miR-base. Interestingly, we observed a 1.4-fold increase in the miR-1896, which has a predicted binding site in the UTRs of several ion transporters including Slc5a11, Slc22a1, Slc8a2, and Slc9a3r2 (NHERF2, https://www.targetscan.org/cgi-bin/targetscan/mmu_80/view_gene.cgi?rs=ENSMUST00000019684.6&taxid=10090&members=miR-1896&showcnc=1&shownc=1&shownc_nc=1&showncf1=1&showncf2=1&subset=1, (accessed on 31 January 2024)). The miRNA-1962 decreased by about 60% in the old mice and has a predicted binding site on position 840–847 in the UTR of dynamin (https://www.targetscan.org/cgi-bin/targetscan/mmu_80/view_gene.cgi?rs=ENSMUST00000113352.3&taxid=10090&members=miR-485-5p/1962&showcnc=1&shownc=1&subset=1, (accessed on 31 January 2024)), a structural protein, that has been shown to bind NHERF1 [24].

The miRNA candidates that might be regulated by aging associated with NHERF1^−/−^ were identified. These miRNAs were used to identify cellular signaling pathways enriched by target genes of the miRNAs. To determine NHERF1- and age-dependent changes in the miRNA profile, we analyzed the data between young and old NHERF1^−/−^ mice. As shown in Table 4, 7 miRNAs increased in only NHERF1^−/−^ old mice as compared to young mice. We also observed a decreased expression in 43 miRNAs. One of the miRNAs that decreased was miRNA-153. miR-153 has a strong conserved binding site on position 1800–1806 of transcription factor Nuclear Factor of Activated T-Cells (NFATc3) UTR of NFATc3 and NFATc2 (https://www.targetscan.org/cgi-bin/targetscan/mmu_80/view_gene.cgi?rs=ENSMUST00000109308.1&taxid=10090&members=miR-153-3p&showcnc=0&shownc=0&subset=1, (accessed on 31 January 2024)).

Differentially expressed miRNAs from each comparison from Table 4 were then used to identify KEGG pathways enriched in miRNA-target genes. KEGG pathway enrichment analysis was performed using mirPath (v3.0, DIANA Tools) with microT-CDS (microT threshold > 0.95, *p*-value < 0.05) in the mouse database (Supplementary Table S2). Eleven cellular signaling pathways (KEGG) were identified from enrichment analysis of miRNA-target genes (Table 5). All the miRNAs associated with these signaling pathways decreased in NHERF1^−/−^ old mice, indicating the activation of the translational process of their target genes.

**Table 5:** Cellular signaling pathways associated with miRNAs changed by aging in NHERF1^−/−^ mice.

KEGG Pathway	*p*-Value	Number of Target Genes of miRNAs	Number of miRNAs
Wnt signaling pathway	0.00054352	30	9
Estrogen signaling pathway	0.00068162	21	10
Neurotrophin signaling pathway	0.00134064	26	10
MAPK signaling pathway	0.00272778	44	12
GnRH signaling pathway	0.00426589	20	9
Oxytocin signaling pathway	0.00426589	31	12
B cell receptor signaling pathway	0.00836808	17	10
cAMP signaling pathway	0.00949667	36	12
cGMP-PKG signaling pathway	0.01397984	32	12
T cell receptor signaling pathway	0.01584336	21	9
FoxO signaling pathway	0.02785748	27	8

139 component genes of the signaling pathways targeted by miRNAs are listed in Supplementary Table S3. Expression of the signaling components along the kidney tubule segments (TPM values, Knepper lab database, [25]) is also shown in the table for comparative reasons.

**Figure 1:**
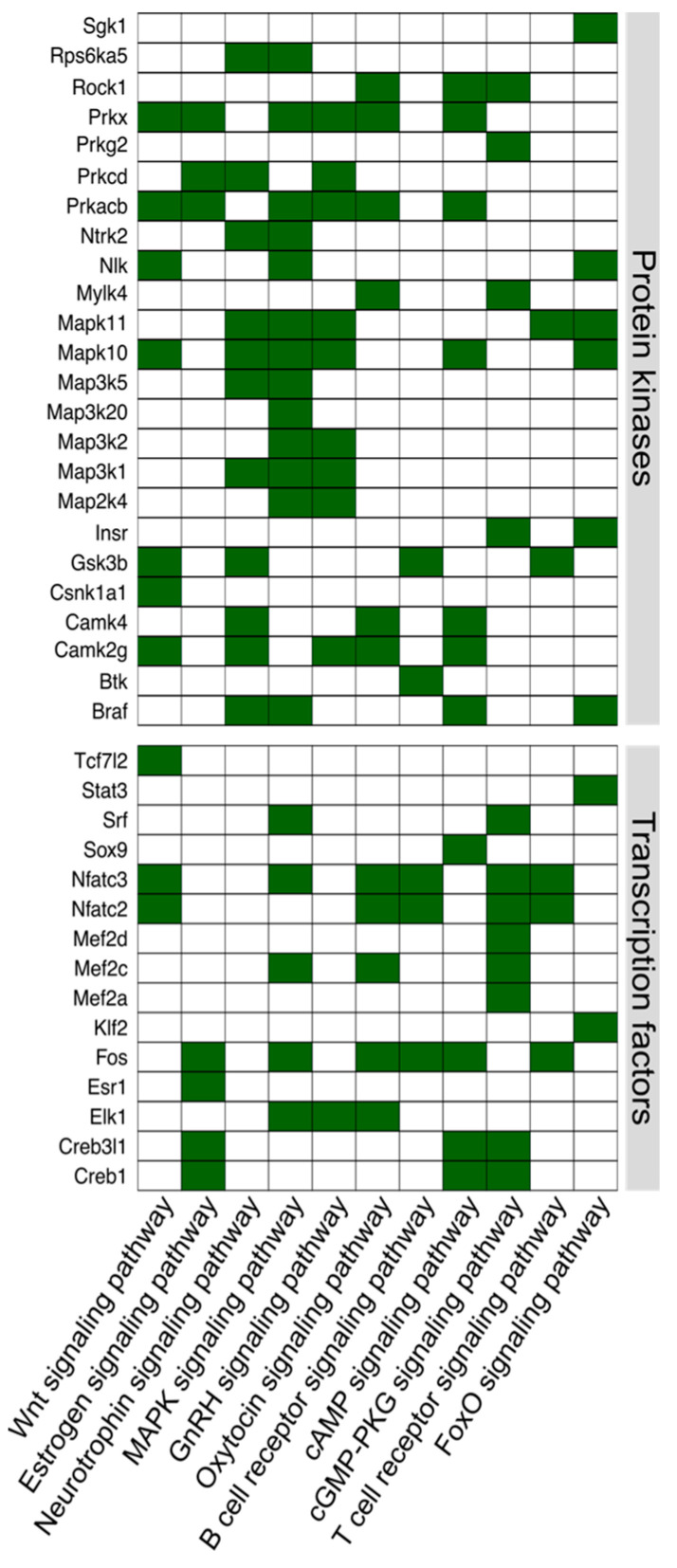
TFs and protein kinases from cellular signaling pathways targeted by miRNAs identified in Table 5. miRNAs differentially expressed in aged NHERF1^−/−^ mouse kidney and their mRNA targets identified from KEGG pathway analysis in Table 5 were plotted by function: Protein Kinases and Transcription Factors. This Figure shows major NHERF1-dependent pathways affected during aging.

As shown in Figure 1, components of Wnt and estrogen signaling pathways are strongly associated with miRNAs changed by the loss of NHERF1 in old mice. Among the components of the Wnt signaling pathway, there are 3 transcription factors (NFATc2, NFATc3, and Tcf7l2) and 7 protein kinases (Csnk1a1, Prkacb, Gsk3b, Camk2g, Nlk, Prkx, and Mapk10). Among the components of the estrogen signaling pathway, there are 4 transcription factors (Creb3l1, Creb1, Esr1, and Fos) and 3 protein kinases (Prkacb, Prkx, and Prkcd). Because Wnt signaling is involved in the regulation of blood pressure [26,27,28] and cytokine expression [29,30,31], we further analyzed the expression of two transcription factors, NFATc2 and NFATc3, within the Wnt signaling cascade that are known to regulate cytokine IL-6 [32,33].

Our data identified a decrease in miRNA153 as an NHERF1-dependent miRNA in aging; miRNA153 is known to regulate the expression of NFATc2 and NFATc3. We therefore examined the nuclear expression of NFATc2 in the WT and NHERF1^−/−^ kidneys. The expression of NFATc2 was validated by the increase in the expression of NFATc2 in the nuclear fraction isolated from kidneys of old WT and NHERF1^−/−^ mice by western blotting. As shown in Figure 2, the expression of NFATc2 significantly increased in the nuclear fraction of kidneys from the NHERF1^−/−^ mice as compared to the WT mice.

**Figure 3:**
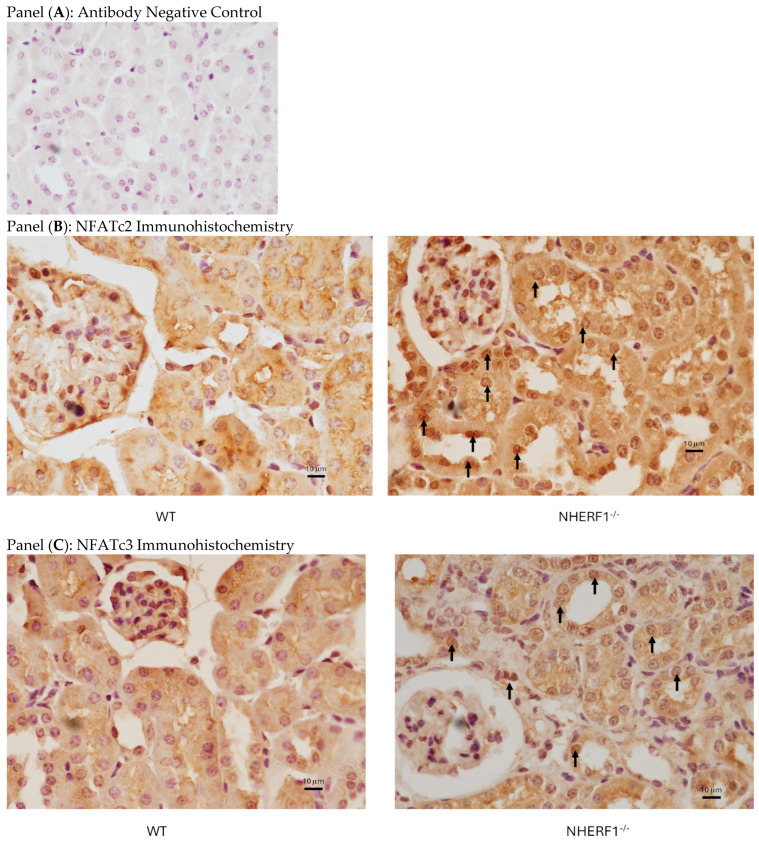
Expression of NFATc2 and NFATc3 in kidneys. Representative image of kidneys from 18–24-month-old WT (**left panels**) or NHERF1^−/−^ (**right panels**) mice (*n* = 6, 3 males and 3 females in each group) analyzed by immunohistochemistry for expression of NFATc2 (panel (**B**)) and NFATc3 (panel (**C**)). Panel (**A**) is a negative control for the antibodies. Arrows show expression staining for NFATc2 (**B**) and NFATc3 (**C**) in the nuclei.

The expression of NFATc2 and NFATc3 was confirmed by immunohistochemistry. As shown in Figure 3, diffuse cellular expression of both NFATc2 (Panel B) and NFATc3 (Panel C) was observed in the cytoplasm of the old WT mice. There was a higher expression of both NFATc2 and NFATc3 in the nuclei of the renal tubular cells in the old NHERF1^−/−^ mice (arrows).

### Expression of Cytokines in the Kidneys of Old WT and NHERF1^−/−^

To determine whether NHERF1-dependent decrease in miRNA-153 and an increase in nuclear expression of NFATc2 and NFATc3 will increase expression of known proinflammatory cytokines involved in chronic kidney disease, viz., IL-1, IL-6, IL-17, MCP-1, and TGFα, we measured the expression of cytokines in the kidney homogenates by ELISA. As shown in Figure 4, expression of IL-17A and MCP1 was significantly higher in the kidneys from the WT old mice as compared to the young WT mice. Expression of IL1β, IL6, and IL-17A was significantly higher in the young NHERF1^−/−^ mice as compared to their age-matched WT mice. Expression of IL-1β, IL-6, IL-17A, MCP-1, and TGFα significantly increased in the kidneys of the old NHERF1^−/−^ mice as compared to the age-matched WT mice and from the young NHERF1^−/−^ mice. In contrast, the expression of IL-10, an anti-inflammatory cytokine did not change between the groups.

## 4. Discussion

Age-related renal impairment is a growing clinical problem [34]. Previously we demonstrated increased NHERF1 expression in the kidneys of FBN rats during aging that is associated with age-related, salt-sensitive hypertension [5,7]. Here, we performed an observational study to determine the effects of NHERF1 expression in aging on epigenetic changes by miRNA-regulated networks. We tested this hypothesis in a NHERF1^−/−^ mouse model and examined miRNA-mediated gene regulation of proinflammatory cytokines in aging.

miRNAs have been shown to regulate diverse physiological functions including cell growth, apoptosis, and metabolism through regulation of mRNA stability and translation [10,11]. miRNAs are also involved in various diseases including hypertension [14,35], cardiovascular and kidney diseases [15,35,36], and diabetes [37]. Aging is a normal but complex physiological phenomenon that leads to renal impairment [34]. Studies have shown a role for miRNAs in the regulation of aging [34]; however, very few studies have been carried out to understand the role of miRNAs in the aging kidney [38]. Kwekel et al. [2] identified changes in total miRNAs in aging male and female F344 rats. They found that 17 miRNAs that regulate hyperplasia, proliferation, and cancer are overrepresented in aging kidneys. These included miR-363, miR-181a, and miR-18. They reported 22 distinct miRNAs that increased with age. These included members of the miRNA-29 family and miRNA-34a that regulate DNA methylation and p53-related apoptosis.

Our results similarly identified reduced miR-181a in the kidneys of the older mice as compared to the young mice. Additionally, our data showed that miR-181a further decreased in the kidneys of aged NHERF1^−/−^ mice as compared to aged WT (Supplemental data). Similar to reports from Kwekel et al. [2,39], our data showed that age effects were predominant over sex effects in regulating miRNA expression in the kidney. Therefore, we combined the data from both male and female mice for analysis. Pathway analysis of the data showed that 13 miRNAs were age dependent. The major pathways associated with age were involved in GABAergic synapses, proteoglycans in cancer, cell adhesion molecules, glycosphingolipid metabolism, and signaling pathways that regulate the pluripotency of stem cells.

Eleven miRNAs changed exclusively in the kidneys of WT mice. These included miRNA-1927 and miR-1966, which increased with age, while miR-1905, miR-1954, miR-1957, miR-217, miR-465a, miR-466, miR-504, and miR-702 decreased. Pathway analysis showed that these miRNAs are involved in morphine addiction, nicotine addiction, hematopoietic cell lineage, oocyte meiosis, SNARE interactions in vesicular transport, and thyroid hormone synthesis.

In the NHERF1^−/−^ mice, 34 miRNAs changed significantly including miR-690 and miR-1960 (increased), while miR-184, miR-153, miR-127, and miR-1932 decreased in the old NHERF1^−/−^ mice compared to the young mice. These were found to be involved in the regulation of the Wnt signaling pathway, estrogen signaling pathway, cAMP and cGMP signaling pathways, and MAPK signaling pathways. Wnt signaling, which regulates numerous downstream pathways, has been demonstrated to change in rat kidneys depending on the severity of hypertension [26,40] and plays a major role in kidney injury and repair [40]. The transcription factors (TFs) NFATc2, NFATc3, and TCF7 are regulated by Wnt signaling. The 3′-UTRs of NFATc2 and NFATc3 are targets of miRNA-153, and miR-669 has a conserved binding site on position 139–146 of the TCF7 3′ UTR, suggesting that these miRNAs target these TFs. Our data showed a decrease in both miR-153 (NHERF1-dependent) and miRNA-669 (age-dependent) in the kidneys of aging mice, suggesting that NFATc2, NFATc3, and TCF7 expression increased in aging mice. Indeed, our histological and western blot data confirmed this hypothesis.

NFATc2 and NFATc3 [41] play an important role in the inducible expression of cytokine genes in T-cells. They are members of the multiprotein component of the NFATC transcription complex. NFATc2 and NFATc3 are cytoplasmic phosphoproteins when inactive. Upon dephosphorylation by calcineurin A, they bind with other members of the NFAT family, translocate to the nucleus, and bind DNA to induce the transcription of IL-2, IL-3, IL-4, IL-6, TNF-α, and GM-CSF [41,42,43,44,45,46,47,48,49,50]. Low levels of expression of NFATc2 and NFATc3 have been reported in kidneys from older compared to younger individuals (https://www.proteinatlas.org/ENSG00000101096-NFATC2/tissue/kidney#imid_2647772, https://www.proteinatlas.org/ENSG00000072736-NFATC3/tissue/kidney, (accessed on 31 January 2024) [51]). Both IL-1b and IL-6 are elevated in several forms of inflammatory chronic kidney diseases, including diabetic kidney disease [52,53,54,55]. Taken together, the data presented here suggest a link between NHERF1-dependent decrease in miR-153 and an increased nuclear expression of NFATc2 and/or NFATc3 in renal tubular cells. NFATc2 and/or NFATc3 may then mediate increased expression of proinflammatory cytokines in the kidneys and can potentially increase susceptibility to kidney injury in older animals as compared to younger animals [9,56,57].

The limitations to our study include, data presented here used whole kidneys, are observational and correlative, and require confirmation. Future studies using overex-pression and knockdown of miR-153 in in vitro kidney cell models and in vivo rodent models are necessary to examine the interaction between miR-153 and NFAT mRNAs associated with this cellular pathway.

## 5. Conclusions

In summary, we report for the first-time changes in miRNA expression profiles in mouse kidneys with age that are dependent upon NHERF1 expression. A limitation of this work is that pathological changes in the kidney during aging are more prominent in the renal cortex. Our laboratory previously demonstrated increased oxidative stress in kidneys from NHERF1^−/−^ mice and in opossum kidney cells with decreased NHERF1 expression [9]. Oxidative stress has been shown to decrease miR-153 expression in HUVEC cells [58]. Taken together, our data may suggest that the decrease in miR-153 expression in the kidneys of older NHERF1^−/−^ mice as compared to young mice may be due to an increase in oxidative stress. Decreased miR-153, potentially targeting NFATc2 and NFATc3 mRNAs, may cause increased expression and/or nuclear translocation of NFATc2 and/or NFATc3 resulting in an increase in inflammatory cytokine expression as detected here. The data presented here are observational, and further studies are required to extend these observations on direct functional interactions between miR-153, NFATc2, and NFATc3, and increased expression of proinflammatory cytokines to understand the molecular mechanisms of aging kidneys.

## Figures and Tables

**Table 1:** Changes in miRNA profile between young (2–4-month-old) WT and NHERF1^−/−^ mice.

Name	Accession	Mean NHERF1^−/−^	SD NHERF^−/−^	Mean WT	SD WT	*t*-Test	NHERF1^−/−^/WT
mmu-miR-691	MI0004659	4.291	0.916	5.411	0.579	0.0259	0.793
mmu-miR-291b-5p	MI0003539	2.698	0.865	3.672	0.590	0.0371	0.735
mmu-miR-18b	MI0005483	2.498	1.088	3.540	0.264	0.0465	0.706

**Table 2:** Changes in miRNA profile between old (18–24-month-old) WT and NHERF1^−/−^ mice.

Name	Accession	Mean NHERF1^−/−^	SD NHERF1^−/−^	Mean WT	SD WT	*t*-Test	NHERF1^−/−^/WT
mmu-miR-684	MIMAT0003462	2.994	0.148	3.759	0.424	0.0103	0.797
mmu-miR-380-5p	MIMAT0000744	3.141	0.331	3.970	0.789	0.0501	0.791
mmu-miR-675-3p	MIMAT0003726	2.598	0.433	3.287	0.607	0.0492	0.790
mmu-miR-293	MIMAT0000371	3.260	0.304	4.145	0.310	0.0194	0.787
mmu-miR-92b	MIMAT0004899	2.873	0.597	3.684	0.256	0.0380	0.780
mmu-miR-539	MIMAT0003169	3.991	0.474	5.151	0.553	0.0127	0.775
mmu-miR-466k	MIMAT0005845	3.060	0.538	3.999	0.648	0.0294	0.765
mmu-miR-1894-3p	MIMAT0007878	2.600	0.400	3.489	0.368	0.0463	0.745
mmu-miR-184	MIMAT0000213	2.614	0.864	3.570	0.675	0.0325	0.732
mmu-miR-188-3p	MIMAT0004541	2.689	1.152	3.847	0.366	0.0392	0.699
mmu-miR-409-3p	MIMAT0001090	2.146	0.867	3.098	0.337	0.0274	0.693
mmu-miR-491	MIMAT0003486	2.807	0.641	4.157	0.351	0.0339	0.675
mmu-miR-669m	MIMAT0009419	1.727	0.571	2.896	0.208	0.0053	0.596

**Table 3:** Changes in miRNA profile between young (2–4-month-old) and old (18–24-month-old) WT mice.

Name	Accession	Mean Old	SD Old	Mean Young	SD Young	*t*-Test	Old/Young
mmu-miR-1186	MIMAT0005836	6.313	0.607	3.634	0.473	0.0001	1.737
mmu-miR-1896	MIMAT0007873	5.695	0.633	4.045	0.658	0.0034	1.408
mmu-miR-375	MIMAT0000739	7.191	0.445	5.278	0.229	0.00003	1.363
mmu-miR-1966	MIMAT0009439	5.763	0.215	4.352	0.310	0.0001	1.324
mmu-miR-1927	MIMAT0009390	5.038	0.252	3.827	0.529	0.0021	1.316
mmu-miR-341	MIMAT0000588	2.388	1.189	3.446	0.393	0.0501	0.693
mmu-miR-466c-5p	MIMAT0004877	2.989	1.132	4.327	0.885	0.0431	0.691
mmu-miR-702	MIMAT0003492	2.429	0.348	3.519	0.247	0.0004	0.690
mmu-miR-1905	MIMAT0007866	2.744	0.518	4.033	0.456	0.0027	0.680
mmu-miR-678	MIMAT0003452	3.494	0.728	5.135	0.304	0.0012	0.680
mmu-miR-504	MIMAT0004889	2.675	1.165	3.972	0.315	0.0230	0.674
mmu-miR-465a-5p	MIMAT0002106	2.592	0.626	3.850	0.454	0.0049	0.673
mmu-miR-217	MIMAT0000679	2.445	1.071	3.654	0.503	0.0292	0.669
mmu-miR-1954	MIMAT0009425	2.445	1.071	3.660	0.382	0.0241	0.668
mmu-miR-1957	MIMAT0009430	3.172	0.577	4.767	0.605	0.0026	0.665
mmu-miR-466d-3p	MIMAT0004931	3.453	0.371	5.434	0.459	0.0001	0.636
mmu-miR-434-5p	MIMAT0001421	3.042	0.821	4.795	0.636	0.0042	0.635
mmu-miR-343	MIMAT0004868	2.429	0.782	3.901	0.551	0.0063	0.623
mmu-miR-1902	MIMAT0007863	4.210	1.087	7.383	0.744	0.0006	0.570
mmu-miR-1947	MIMAT0009413	2.250	0.500	3.951	0.219	0.0001	0.569
mmu-miR-377	MIMAT0000741	3.113	0.855	5.672	0.418	0.0003	0.549
mmu-miR-1274a	MIMAT0009445	2.282	0.901	4.280	0.216	0.0009	0.533
mmu-miR-1958	MIMAT0009431	2.623	1.845	4.961	0.701	0.0166	0.529
mmu-miR-1962	MIMAT0009435	2.042	0.748	4.513	0.416	0.0002	0.453

**Table 4:** Changes in miRNA profile between young (2–4-month-old) and old (18–24-month-old) NHERF1^−/−^ mice.

miRNAs	miRNA ID	Fold Change in Old vs. Young in WT	*p*-Value of Old vs. Young in WT	Fold Change in Old vs. Young in KO	*p*-Value of Old vs. Young in KO	Aging Dependent *	WT Only. **	Aging Associated with KO. ***
mmu-miR-1186	MIMAT0005836	1.737	7.05 × 10^−5^	1.756	7.93 × 10^−5^	Yes		
mmu-miR-690	MIMAT0003469			1.354	7.07 × 10^−3^			Yes
mmu-miR-1960	MIMAT0009433			1.307	6.04 × 10^−3^			Yes
mmu-miR-1274a	MIMAT0009445	0.533	9.14 × 10^−4^	0.490	1.69 × 10^−3^	Yes		
mmu-miR-1839-3p	MIMAT0009457			0.698	5.33 × 10^−4^			Yes
mmu-miR-3471	MIMAT0015642			0.696	4.26 × 10^−2^			Yes
mmu-miR-466d-5p	MIMAT0004930			0.693	1.46 × 10^−3^			Yes
mmu-miR-511	MIMAT0004940			0.690	2.21 × 10^−3^			Yes
mmu-miR-380-5p	MIMAT0000744			0.689	7.76 × 10^−3^			Yes
mmu-miR-1896	MIMAT0007873	1.408	3.36 × 10^−3^	1.398	4.57 × 10^−3^	Yes		
mmu-miR-1902	MIMAT0007863	0.570	6.18 × 10^−4^	0.591	5.11 × 10^−4^	Yes		
mmu-miR-1905	MIMAT0007866	0.680	2.68 × 10^−3^				Yes	
mmu-miR-1927	MIMAT0009390	1.316	2.09 × 10^−3^				Yes	
mmu-miR-376c	MIMAT0003183			0.688	1.27 × 10^−2^			Yes
mmu-miR-1970	MIMAT0009444			0.683	1.48 × 10^−4^			Yes
mmu-miR-1947	MIMAT0009413	0.569	1.14 × 10^−4^	0.605	9.79 × 10^−3^	Yes		
mmu-miR-1954	MIMAT0009425	0.668	2.41 × 10^−2^				Yes	
mmu-miR-1957	MIMAT0009430	0.665	2.57 × 10^−3^				Yes	
mmu-miR-1958	MIMAT0009431	0.529	1.66 × 10^−2^	0.532	1.84 × 10^−2^	Yes		
mmu-miR-1946b	MIMAT0009443			0.680	1.13 × 10^−2^			Yes
mmu-miR-1962	MIMAT0009435	0.453	1.96 × 10^−4^	0.412	2.18 × 10^−3^	Yes		
mmu-miR-599	MIMAT0012772			0.680	2.76 × 10^−3^			Yes
mmu-miR-1966	MIMAT0009439	1.324	5.86 × 10^−5^				Yes	
mmu-miR-1964	MIMAT0009437			0.674	6.48 × 10^−4^			Yes
mmu-miR-297b-5p	MIMAT0003480			0.671	2.46 × 10^−3^			Yes
mmu-miR-217	MIMAT0000679	0.669	2.92 × 10^−2^				Yes	
mmu-miR-483	MIMAT0004782			0.657	6.81 × 10^−3^			Yes
mmu-miR-92b	MIMAT0004899			0.651	5.07 × 10^−3^			Yes
mmu-miR-341	MIMAT0000588	0.693	5.01 × 10^−2^				Yes	
mmu-miR-343	MIMAT0004868	0.623	6.35 × 10^−3^	0.617	5.42 × 10^−3^	Yes		
mmu-miR-876-5p	MIMAT0004854			0.650	7.23 × 10^−4^			Yes
mmu-miR-139-3p	MIMAT0004662			0.643	2.19 × 10^−3^			Yes
mmu-miR-375	MIMAT0000739	1.363	3.28 × 10^−5^	1.414	6.66 × 10^−3^	Yes		
mmu-miR-1186b	MIMAT0015644			0.641	1.23 × 10^−2^			Yes
mmu-miR-377	MIMAT0000741	0.549	2.89 × 10^−4^	0.647	1.19 × 10^−3^	Yes		
mmu-miR-224	MIMAT0000671			0.641	5.92 × 10^−4^			Yes
mmu-miR-491	MIMAT0003486			0.633	8.33 × 10^−3^			Yes
mmu-miR-434-5p	MIMAT0001421	0.635	4.24 × 10^−3^	0.685	1.97 × 10^−2^	Yes		
mmu-miR-465a-5p	MIMAT0002106	0.673	4.94 × 10^−3^				Yes	
mmu-miR-466c-5p	MIMAT0004877	0.691	4.31 × 10^−2^				Yes	
mmu-miR-466d-3p	MIMAT0004931	0.636	1.09 × 10^−4^	0.556	2.83 × 10^−4^	Yes		
mmu-miR-711	MIMAT0003501			0.618	1.78 × 10^−3^			Yes
mmu-miR-1894-3p	MIMAT0007878			0.617	1.01 × 10^−3^			Yes
mmu-miR-495	MIMAT0003456			0.612	1.32 × 10^−2^			Yes
mmu-miR-1969	MIMAT0009442			0.607	1.25 × 10^−2^			Yes
mmu-miR-411	MIMAT0004747			0.593	2.43 × 10^−4^			Yes
mmu-miR-504	MIMAT0004889	0.674	2.30 × 10^−2^				Yes	
mmu-miR-466k	MIMAT0005845			0.589	2.82 × 10^−4^			Yes
mmu-miR-184	MIMAT0000213			0.586	4.16 × 10^−3^			Yes
mmu-miR-1932	MIMAT0009395			0.580	3.09 × 10^−3^			Yes
mmu-miR-369-5p	MIMAT0003185			0.563	2.14 × 10^−2^			Yes
mmu-miR-669m	MIMAT0009419			0.549	2.35 × 10^−3^			Yes
mmu-miR-678	MIMAT0003452	0.680	1.21 × 10^−3^	0.620	2.90 × 10^−3^	Yes		
mmu-miR-590-3p	MIMAT0004896			0.521	1.66 × 10^−3^			Yes
mmu-miR-702	MIMAT0003492	0.690	4.45 × 10^−4^				Yes	
mmu-miR-153	MIMAT0000163			0.483	1.78 × 10^−2^			Yes
mmu-miR-673-3p	MIMAT0004824			0.467	5.10 × 10^−3^			Yes
mmu-miR-127	MIMAT0000139			0.464	4.38 × 10^−3^			Yes

* “Aging dependent”: commonly changed miRNAs (old versus young) in WT and KO; these are aging-related miRNAs. ** “WT only”: potential miRNA candidates for maintaining WT that might be disrupted by KO. *** “Aging associated with KO”: potential miRNA candidates that play a role in KO; these are candidates regulated by NHERF1 during aging.

## Data Availability

All raw data has been deposited, published on Mendeley data set (https://data.mendeley.com/drafts/xkp9dy9z4f, Reserved https://doi.org/10.17632/xkp9dy9z4f.1), and is publicly available.

## Supplementary Materials

The following supporting information can be downloaded at: https://www.mdpi.com/article/10.3390/biom14091048/s1. File S1: Spplementary Tables. Original images can be found in Figure S1.
